# Communication makes intensive language therapy more efficient: a randomized controlled trial in chronic post-stroke aphasia

**DOI:** 10.1093/braincomms/fcag252

**Published:** 2026-07-03

**Authors:** Friedemann Pulvermüller, Anna-Thekla Jäger, Milena R Osterloh, Johanna Knechtges, Verena Arndt, Anna Sadlon, Marcelo L Berthier, Bettina Mohr

**Affiliations:** Brain Language Laboratory, Department of Philosophy and Humanities, Freie Universität Berlin, Berlin 14195, Germany; Berlin School of Mind and Brain, Humboldt Universität zu Berlin, Berlin 10099, Germany; Einstein Center for Neurosciences Berlin, Berlin 10117, Germany; Brain Language Laboratory, Department of Philosophy and Humanities, Freie Universität Berlin, Berlin 14195, Germany; Max Planck Institute for Human Cognitive and Brain Sciences, Leipzig 04103, Germany; Charité Universitätsmedizin, Berlin 10117, Germany; Brain Language Laboratory, Department of Philosophy and Humanities, Freie Universität Berlin, Berlin 14195, Germany; Einstein Center for Neurosciences Berlin, Berlin 10117, Germany; Brain Language Laboratory, Department of Philosophy and Humanities, Freie Universität Berlin, Berlin 14195, Germany; Brain Language Laboratory, Department of Philosophy and Humanities, Freie Universität Berlin, Berlin 14195, Germany; Brain Language Laboratory, Department of Philosophy and Humanities, Freie Universität Berlin, Berlin 14195, Germany; Cognitive Neuroscience and Aphasia Unit, Faculty of Psychology and Speech Therapy, University of Malaga, 29071 Malaga, Spain; Brain Language Laboratory, Department of Philosophy and Humanities, Freie Universität Berlin, Berlin 14195, Germany; Center for Neuropsychology and Intensive Language Therapy, Berlin 10629, Berlin, Germany

**Keywords:** communication, depression, intensive speech-language therapy, neurorehabilitation, post-stroke aphasia

## Abstract

Speech-language therapy is effective in chronic post-stroke aphasia if it is delivered in an intensive fashion. The study investigated whether intensive communicative therapy by practicing everyday language use (e.g. requesting objects and proposing actions) leads to better outcomes than equally intensive conventional treatment (e.g. picture naming). A randomized controlled trial compared intensive conventional speech-language therapy to a social-communicative treatment, called Intensive Language Action Therapy, a further development of Constraint-Induced Aphasia Therapy. Forty-four chronic post-stroke aphasia patients randomized to one of these treatments received 2 weeks, 5 days/week, for 2.5 h/day of therapy. Primary outcome measure was an established clinical language battery. To address any changes in mood, patients’ depressive symptoms were assessed using Beck’s Depression Inventory. A significant interaction [*F*(1,42) = 7.42; *P* = 0.009] revealed stronger language improvements across Intensive Language Action Therapy as compared to conventional therapy [*t*(22) = 3.93, *P* < 0.001 versus *t*(20) < 1.5, non-significant]. Likewise, depression scores after Intensive Language Action Therapy were significantly reduced. These results show that different types of intensive speech-language therapy can improve language skills of chronic post-stroke aphasia patients to different degrees. Intensive Language Action Therapy led to significantly stronger improvements on a clinical language test battery than equally intensive conventional therapy. Furthermore, our data suggest that patients’ symptoms of depression improved to different degrees depending on the type of therapy delivered.

## Teaser

Pulvermüller *et al*. report that intensive communicative speech-language therapy leads to greater improvements of clinical language performance in chronic post-stroke aphasia than matched conventional language therapy. A concordant change was present for depressive symptoms, which diminished more with communicative therapy. The results advocate for intensive practice of behaviourally relevant communicative interaction in the therapy of chronic stroke-related aphasia.

## Graphical Abstract

**Graphical Abstract:**
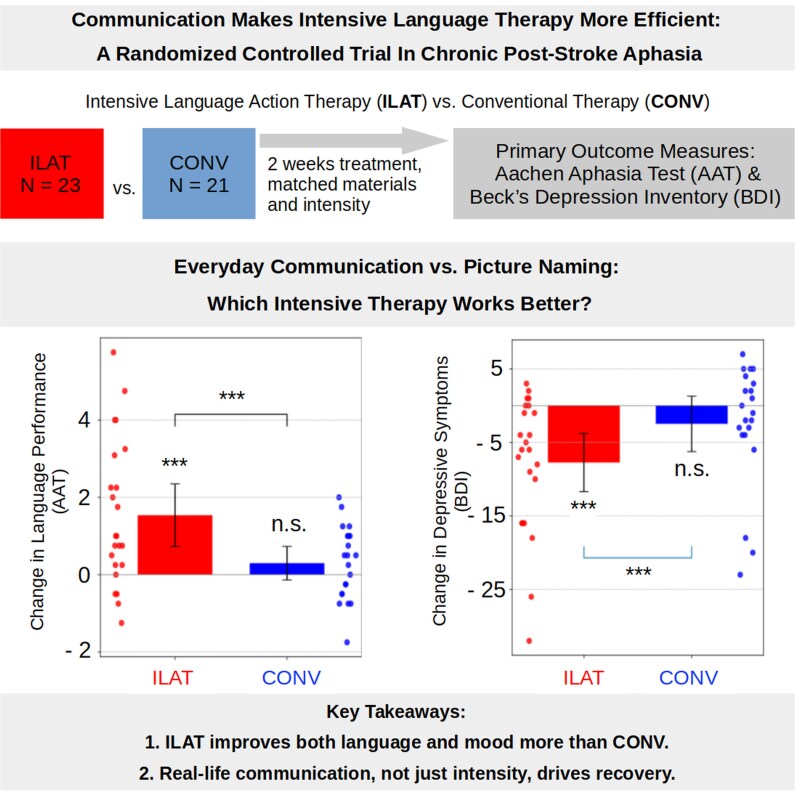

## Introduction

Behavioural speech-language therapy (SLT) is an effective tool for improving language performance in patients with chronic post-stroke aphasia (PSA).^1-5^ But which factors are most relevant for achieving such success? Therapy researchers highlighted several such putative factors, but just one of these, the *amount and intensity* with which SLT is provided, so far received strong experimental support. In particular, intensive regimes of at least 5 h per week, but not sparser schedules, led to reliable improvements in chronic PSA,^2,5,6^ although a similar intensity effect was not confirmed in acute aphasia.^7,8^ The strongest support for better outcomes with relatively higher amounts and intensities of treatment came from randomized controlled trials. Prolonging the therapy interval may further increase the degree of improvement in chronic aphasia, whereas further increasing intensity—for example from 6 to 12 h per week—did not.^9^ Contrasting with the well-documented effects of therapy amount and intensity, no agreement exists regarding other factors that could influence therapy outcome. Recent reviews put that different traditional and modern therapeutic approaches are equally effective in improving language and communication given they are delivered with the same amount and intensity.^2,5,10^ This suggests that, within certain limits, ‘more helps more’, no matter what kind of SLT is delivered and what exactly the patients practice.

The language tasks of classic SLT focus on the production or comprehension of specific utterances or linguistic structures (e.g. speech sounds, words and sentences). Typical examples of such tasks are confrontation naming, where patients are shown real or depicted objects and are asked to produce a ‘name’ or description for each, and language comprehension, whereby the patients’ task is to select and point to an object or picture matching the meaning of an utterance. The aim is to produce, classify and understand an utterance, also applying to linguistic tasks, e.g. phoneme distinction, selecting items from a semantic field or sentence completion. Although some utterance-centred tasks may be used in adult–infant interaction and second-language teaching, these play a minor role, if one at all, in day-to-day communicative interaction between adults. Clearly, finding the right words is essential for successful communication^11^; however, in adult communication, words and utterances, proper names and category labels are rarely used just to correctly name an object. Language use commonly serves further reaching goals or purpose, such as obtaining something from a partner, making a proposal for joint action or reporting past experiences.^12^ This discrepancy regarding language function led to suggestions to replace the utterance-centred, structural tasks of classic SLT by communicative and social-interactive ways of using language.^13-16^ If improving language performance in communication is the main therapeutic goal, social-communicative therapies may appear preferable.

Recent novel arguments in favour of communicative therapies come from neuroscience research in healthy individuals demonstrating stronger brain activity when language is used for communication than when it is applied with the only purpose of utterance production or comprehension. For example, the processing of the same word in the utterance-based naming task is accompanied by reduced brain activity compared with the same form being used to make a request, a communicative function very relevant in daily life^17-21^ in language comprehension and production.^22,23^ The areas showing stronger neuronal activity for requests than naming are associated with additional engagement of linguistic, action-related and visual-attentional processes for requests. The fact that cortical activity is stronger in request than naming contexts, even if exactly the same utterances are applied, suggests that the communicative function of requesting may have a relatively greater potential to spark neural reorganization processes.^15,24^ Neuroimaging findings therefore motivate a closer look at studies comparing classic structural language therapy with communicative methods.

Pioneering randomized controlled SLT studies compared the effect of utterance-centred language tasks with that of conversation groups and reported comparable improvements not only in acute stroke, where spontaneous remission is common and may thus mask differences in therapy effectiveness, but in the chronic stage, after the first 6 months following disease onset.^25^ Similarly, a randomized controlled trial (RCT) performed in acute PSA patients showed comparable improvements for linguistic versus communicative treatment.^26^ Contrasting with ‘free’ conversation, some communicative-pragmatic therapies used constraints for tailoring language use to the patients’ communicative deficits and needs.^12,14,27-29^ One such method, Constraint-Induced Aphasia (or Language) Therapy (CIAT) and its further developed form called Intensive Language Action Therapy (ILAT), was tested in several RCTs, which demonstrated its beneficial effect on language in chronic PSA^1,30-33^ (for review, see^34^), but not in acute or subacute aphasia.^8,35,36,37^ The first study reporting better outcomes of ILAT/CIAT than for classic structural therapies^1^ was affected by a frequency confound (15 versus 7–8 h/week) so that the observed efficacy differences between therapies might be attributed to the frequency factor.^2,5^ However, a more recent pilot cross-over RCT controlled for the intensity, duration and amount of therapy applied (and likewise for verbal and non-verbal therapy content) and suggested more favourable outcomes after communicative treatment targeting requests compared with intensive naming as structural control.^32^ Still, the small number of patients tested in this cross-over pilot alongside its complex design call for reinvestigating the issue with a larger cohort.

An observation of potentially great clinical relevance was that, over and above its beneficial effects on language, intensive communicative aphasia therapy led to significant improvements of patients’ mood and emotional well-being. In particular, chronic PSA patients, who frequently suffer from co-morbid neuropsychiatric symptoms, such as depression,^38-40^ showed significant reduction of depressive symptoms across a short ILAT interval of 2 weeks.^41-43^ Since the presence of additional affective symptoms may compromise functional recovery and traditional psychotherapy cannot be applied to people with aphasia (PWA),^44,45^ the present study put an additional focus on systematic monitoring symptoms of depression across aphasia therapy.

The present registered parallel-group semi-blinded RCT was performed to clarify whether communicative and conventional SLT lead to different degrees of improvement of language and depressive symptoms. Chronic PSA patients were randomly assigned to one of two types of SLT, ILAT^46^ or conventional Intensive SLT (CONV) focusing on practicing language structures, i.e. words and larger utterances.^1,15,47^ Both therapies were delivered in groups of three patients plus one therapist using the same picture sets and identical or matched verbal target expressions. Therapy was administered for 2 weeks, 12.5 h per week. Before and after therapy, language and communication tests were administered and, in addition, symptoms of depression were assessed. Long-term follow-up assessment was not addressed, as one of our earlier studies had demonstrated that short intervals of ILAT led to long-term language improvements lasting for 1–2 years.^48^ Outcome predictions motivated by the above-discussed research were as follows: (i) both intensive therapy regimes improve language and communication across the 2-week treatment interval; (ii) improvements in language and communication are more pronounced for the social-communicative method than for the utterance-centred structural approach; and (iii) improvements of depressive symptoms are present after social-communicative, but not after conventional, SLT.

## Patients and methods

### Participants

The parallel-group RCT was designed to compare the efficacy of ILAT and CONV SLT based on clinical assessment performed within two working days before/after therapy. The study was approved by the Ethics Committee of Charité University Hospital Berlin, Campus Mitte [EA1/384/16]. A power calculation based on previous research had revealed that, in order to show a difference between the two therapy types on the primary outcome language measure at an effect size of 0.8 and a power of 0.8, it is necessary to include at least 42 participants. Recruiting was terminated after this number was reached. After advertising the study in newspapers and on the internet, we received a continuous flow of expressions of interest from PSA patients, their relatives, friends and therapists. Patients were included in the study if they were at least 18 years old, native speakers of German, right-handed, had an ischaemic or haemorrhagic stroke involving the left hemisphere, were diagnosed as aphasic based on the Token Test^49^ and were at least 12 months post-stroke. They were also required to have sufficient attentional, perceptual and motor skills to partake in testing and card game playing and, importantly, to give their informed consent on an informed consent form sent before the start of the study. Exclusion criteria were severe visual or auditory perceptual deficits, pronounced apraxia or agnosia, second stroke or additional neurological disease and withdrawal of informed consent. One hundred forty patients were screened by the therapy manager for some of these in- and exclusion criteria during a short telephone interview and, if found suitable, invited for a 1-h screening session at the Brain Language Laboratory, Freie Universität Berlin, where the study was performed. Fifty-four PSA patients met all inclusion and no exclusion criteria, were included in the study and were assigned to therapy groups, which were numbered according to the order of patient admissions (18 groups).

**Figure 1:**
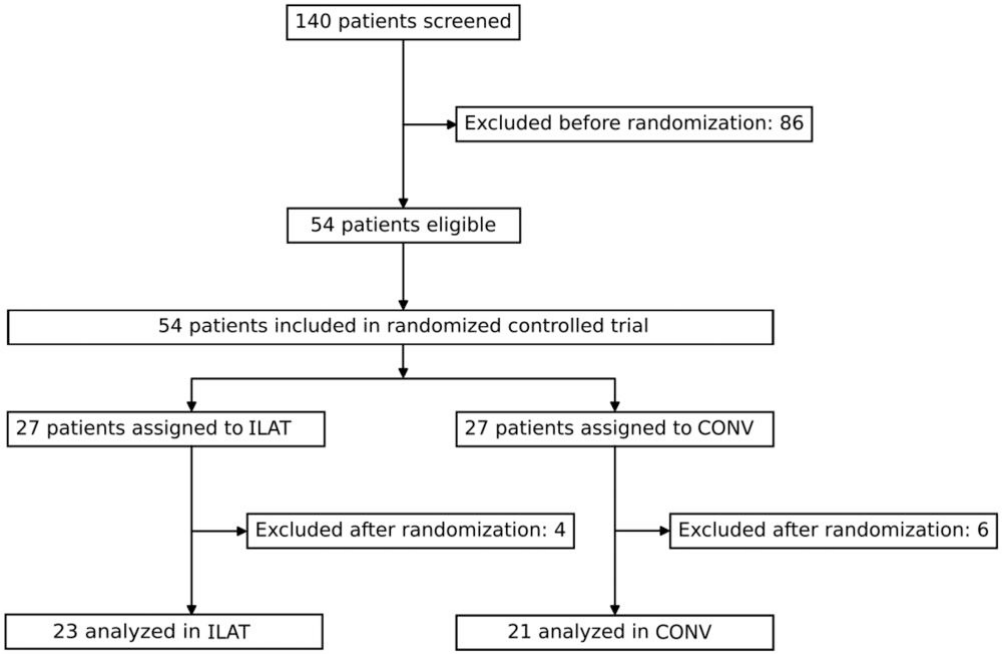
**CONSORT flow diagram.** A total of 140 patients were screened. Fifty-four met the inclusion criteria but not those for exclusion. These were assigned to therapy groups and randomized to ILAT, or intensive conventional, CONV, language therapy. Ten patients had to be excluded after randomization (see Patients and methods and Participants sections). The final analysis included 44 patients (ILAT, 23; CONV, 21).

Therapy groups of three patients each were randomized to one of the therapy methods, ILAT or CONV, by using a computer program with random number generation. A person not involved in testing or therapy generated a sequence of the numbers 0 (for CONV) and 1 (for ILAT). After initial inclusion, group assignment, and before completion of therapy, 10 patients dropped out due to the following incidents fulfilling a previously unmet exclusion criterion: newly obtained medical information indicating an additional neurological disease (second stroke, Parkinson’s disease—one patient each); a transient ischaemic attack during therapy (TIA, one patient); changes in medication prescribed by their neurologist with strong implications for attentional, perceptual and motor performance (one patient); and previous aphasia diagnosis not confirmed by the Token Test carried out at T1 (three patients). Three and four of these seven patients had respectively been assigned to CONV and ILAT. In addition, one group of three patients was excluded because therapy had to be terminated prematurely, unexpectedly, immediately and without final testing at the first COVID-19 lockdown in March 2020. There were no dropouts for other reasons. Thus, 44 participants completed treatment and testing, and their data entered evaluation, 23 of these receiving ILAT and 21 CONV (see Tables 1 and 2 and CONSORT diagram, Fig. 1).

**Table 1:** Clinical and sociodemographic information for each of the 23 chronic PSA patients included in this study and receiving ILAT treatment

Patient ID	Group	Age (y)	Gender	Months since stroke (m)	Years of education (y)	Diagnosis	Severity
1	ILAT	62	2	24	16	G	Severe
2	ILAT	71	1	104	17	W	Moderate
3	ILAT	74	2	25	15.5	A	Mild
4	ILAT	77	2	21	19	G	Severe
5	ILAT	73	2	99	20	B	Moderate
6	ILAT	67	2	183	14	G	Severe
7	ILAT	33	2	18	13	W	Moderate
8	ILAT	45	2	31	20	B	Moderate
9	ILAT	60	1	84	22	A	Mild
10	ILAT	77	2	12	21	A	Mild
11	ILAT	76	1	12	14	W	Moderate
12	ILAT	62	2	47	12	A	Mild
13	ILAT	58	2	30	15.5	B	Moderate
14	ILAT	58	2	25	18.5	A	Mild
15	ILAT	59	2	28	17	B	Moderate
16	ILAT	69	2	12	11	G	Severe
17	ILAT	59	2	33	13	B	Moderate
18	ILAT	46	1	79	15	A	Mild
19	ILAT	54	2	61	13	B	Moderate
20	ILAT	69	2	63	12	A	Moderate
21	ILAT	69	2	18	13	W	Moderate
22	ILAT	57	1	249	13	B	Moderate
23	ILAT	61	2	47	23	G	Severe
MEAN	ILAT	62.43	1.78	56.74	13.28		1.96
SE	ILAT	2.31	0.09	12.14	1.29		0.15
CI	ILAT	[57.65; 67.22]	[1.6; 1.96]	[31.57; 81.91]	[10.61; 15.96]		[1.65, 2.26]

Aphasia diagnoses were provided by the patients’ neurologists and reviewed by a neuropsychologist (B.M.), whereas aphasia severity was calculated as the average of the severities of impairment revealed by the AAT subtests.

Abbreviations: 1, female; 2, male; A, amnesic; B, Broca; CI, confidence interval (95%); G, global; m, months; NC, non-classifiable aphasia; SE, standard error; W, Wernicke; y, years.

**Table 2:** Clinical and sociodemographic information for each of the 21 chronic PSA patients included in this study and receiving CONV treatment

Patient ID	Group	Age (y)	Gender	Months since stroke (m)	Years of education (y)	Diagnosis	Severity
1	CONV	33	1	122	19	W	Moderate
2	CONV	74	2	25	16	G	Severe
3	CONV	59	2	13	16	B	Severe
4	CONV	65	1	34	17	A	Mild
5	CONV	64	2	28	15	G	Severe
6	CONV	72	2	58	13	B	Moderate
7	CONV	62	2	16	16	B	Moderate
8	CONV	42	1	122	11	B	Moderate
9	CONV	51	2	48	15	G	Severe
10	CONV	54	2	39	16	G	Severe
11	CONV	75	1	303	12	W	Moderate
12	CONV	70	2	70	16	G	Severe
13	CONV	51	1	76	13	W	Moderate
14	CONV	33	1	28	19	A	Moderate
15	CONV	74	2	140	19	A	Moderate
16	CONV	61	2	104	12	B	Moderate
17	CONV	56	2	94	13	B	Moderate
18	CONV	64	2	60	18	G	Severe
19	CONV	55	1	141	17	B	Moderate
20	CONV	76	2	19	14	A	Mild
21	CONV	57	2	25	19	B	Mild
MEAN	CONV	59.43	1.67	74.52	15.52		2.19
SE	CONV	2.76	0.11	14.66	0.55		0.15
CI	CONV	[53.67; 65.19]	[1.45; 1.89]	[43.94; 105.11]	[14.38; 16.67]		[1.88, 2.50]
x̅ difference ILAT versus CONV		*t* = 0.84	*t* = 0.84	*t* = −0.93	*t* = −1.6		*t* = −1.15
		df = 42	df = 42	df = 42	df = 42		df = 42
		*P* = 0.41	*P* = 0.4	*P* = 0.35	*P* = 0.13		*P* = 0.26

Aphasia diagnoses were provided by the patients’ neurologists and reviewed by a neuropsychologist (B.M.), whereas aphasia severity was calculated as the average of the severities of impairment revealed by the AAT subtests.

Abbreviations: 1, female; 2, male; A, amnesic; B, Broca; CI, confidence interval (95%); G, global; m, months; NC, non-classifiable aphasia; SE, standard error; W, Wernicke; x̅, sample mean; y, years.

### Behavioural outcome measures

Before (time point T1) and after therapy (T2), all partaking PWA underwent assessment using (i) the *Naming, Repetition, Comprehension* and *Token Test* subtests of the Aachen Aphasia Test (AAT battery^50^); (ii) the Amsterdam Nijmegen Everyday Language Test (ANELT^51^); (iii) the Action Communication Test (ACT^52^); and (iv) the Communicative Activity Log’s (CAL^24^) quantity of communication questionnaire completed by a relative of the participants. Because depression compromises the quality of life of many PSA patients,^38,53,54^ but may be reduced by ILAT [cf hypothesis (v) and refs^41,42^], we also administered (vi) Beck’s Depression Inventory (BDI^55^), a standardized clinical self-rating scale. The present study used the BDI-V,^56^ a simplified 20-item version. Items on the BDI-V are rated on a 6-point Likert scale and contain short, simplified sentences and an additional visualization of symptom severity. As language comprehension was relatively unimpaired in our patients, administration was unproblematic. If necessary, patients were guided by the blinded clinician while making rating, e.g. by reading out loud items or by rephrasing them. The (vii) Montgomery−Åsberg Depression Rating Scale (MADRS^57^), a standardized clinician’s rating scale, was also used. The mean of the four aforementioned subtests of the AAT, here called mAAT, was used as the primary outcome measure for assessing language abilities [see hypotheses (i) and (ii)]. As this study tested an additional prediction about depression reduction [hypothesis (iii)], the well-known depression inventory BDI was chosen as the primary outcome for addressing this hypothesis specifically. All assessments were administered by testers previously trained by a clinical neuropsychologist. Testers and patients were blind to therapy assignment.

### Therapy procedures

Each patient group received 2 weeks of intensive SLT—either ILAT or CONV—for 12.5 h per week delivered in 2.5-h sessions on 10 consecutive working days. The same speech therapists administered both therapies. Each therapy group included three PWA and a therapist; these participants sat around a therapy table, each with picture cards in front of them. These cards showed objects, actions or scenes and were the targets of language use during language games in ILAT and during picture naming and description in CONV. The card set used in a given instance of a game included two copies of each picture card.

ILAT was conducted using language games modelling request and planning conversations.^46,58^ At the start of each game, each player owned 6–8 picture cards whose duplicates were owned by other participants. Barriers on the table prevented players from seeing each other’s cards. In each ‘round’ of the game, the goal of the starting player was to obtain a matching card pair by obtaining the duplicate of one of their own cards. This had to be done by first selecting a card from the own set and by verbal and non-verbal communication with other players. In the request game, the utterances used included an expression referring to the object depicted on the card (‘the cake’, ‘sweet thing’), which could be embedded in carrier phrases (e.g. ‘I want the […],’ ‘Could I please have the […]’). In the planning game, the picture card showed human activities, and players proposed joint activities [using utterances such as ‘(let’s go for a) swim’, ‘(let’s) cook spaghetti’], to which a partner was licensed to agree if they owned the corresponding duplicate. If the addressee indicated that they own the duplicate, the initiator and the addressee compared their selected cards, and in case of a match, the addressee handed over their card and fulfilled the request or agreed to join the activity. If the duplicate was not available, the addressee rejected the request or proposal, and the initiator addressed a different player. In case of card mismatch and thus misunderstanding, the players engaged in conversational repair. After successful completion of a round, the addressee became the initiator of the next round. This was iterated until no cards were left. If a player intended to start a turn but could not find an appropriate utterance or gesture, or if one was used but not understood by other players, the addressed co-player or the therapist provided help (e.g. ‘Would you like a salad?’, ‘Cooking?’), so that these proposals could be accepted or rejected. The objects, actions and scenes shown on the cards and the matching target utterances of a game were adjusted to the partaking patients’ language skills (for details, see^46^). Phonological and semantic constraints were introduced implicitly, by pictures of objects/actions designated by minimal pairs (e.g. ‘deer’, ‘beer’; ‘booking’, ‘looking’) or semantic neighbours (‘lime’, ‘lemon’). While picture cards constrained the utterances applicable for successful communication, the CIAT constraint of discouraging non-verbal language and gesturing was not applied (see evidence reported by^33,59,60^).

Conventional SLT, CONV, was conceived to maximally resemble ILAT in as many ways as possible; the critical difference was the main goal of language use, which was communication and thus realizing communicative goals in ILAT (obtaining an object by requesting it, proposing and agreeing on a joint action), but utterance production and comprehension *per se* in CONV (correctly naming/describing objects or actions, making phonological/semantic distinctions, completing sentences). Participants sat around a table with their cards in front of them, but barriers were absent so that all cards were visible to all. Participants took turns clockwise selecting a card, placing it upside up on a stack in the middle of the table and naming/describing the depicted object, action or scene. In syntactic tasks, target expressions had to be embedded in a carrier phrase of similar length and syntactic complexity as during ILAT (e.g. ‘This is a […]’, ‘The man is […] (preparing spaghetti)’), which the therapist provided initially. Picture cards provided phonological constraints by showing minimal pairs and semantic constraints by including objects/actions from one narrow semantic neighbourhood. Patients had to observe whether or not other players named or described card content correctly. Again, the difficulty level of the target words and the carrier phrases was tailored to the patients’ individual language skills.

Card sets were balanced across therapies so that the same pictures and hence target utterances were used during both types of therapy. In addition, speaking time was equally divided amongst all participating PWA, so that this feature was balanced too. Practicing involved both speaking and spoken language understanding. The card matching procedure in ILAT and the open view on the participants’ card sets during CONV allowed the PWA to assess matches and mismatches between picture cards and any utterances used to refer to their content.

In both types of training, the therapist (i) acted as a model of language use, thereby introducing carrier phrases and referring expressions; (ii) provided instruction and advice (cueing strategies, etc.) whenever helpful; and (iii) motivated participants by giving positive feedback and encouragement. The training materials were designed for the purpose of the current trial. There were 40 card sets, each including 12 picture pairs. For tailoring these sets to individual language skills, the following difficulty levels were available: card sets including items with high, medium and low normalized lemma frequency, items of phonological minimal pairs and items from only one semantic category. Advanced sets included objects only differing in colour, size and/or number or similar actions (e.g. walking and running). Card sets of one difficulty level were matched for mean normalized lemma frequency to ensure that items of each level were similarly challenging (see^46,61,62^).

To ensure accurate application of standard ILAT and CONV SLT methods, two senior scientists experienced with ILAT and CONV supervised each therapist’s performance regularly. In addition, the therapists assured patient compliance with the therapy rules during each game and intervened with correction and modelling in case of deviation from the rules.

### Statistical analyses

Data analysis was conducted using *Jamovi* (version 2.7.12;https://www.jamovi.org).

Repeated-measures analyses of variance (ANOVAs), including the factors *Time* (before versus after therapy, i.e. T1 versus T2) and *Therapy Type* (communicative versus structural, i.e. ILAT versus CONV), were conducted for the primary outcome measures [mAAT for hypotheses (i) and (ii) and BDI for (iii)] and subsequently for all other tests administered (ACT, ANELT, CAL, and MADRS). Significant interactions were further investigated using planned comparison paired-samples *t*-tests. When interaction effects failed significance, Planned comparison *t*-tests were explored to assess the hypothesized presence versus absence of significant effects in ILAT and CONV. Furthermore, to test for any baseline differences in language and depression scores, independent-samples *t*-tests between the groups were conducted for T1 (before therapy) results. In case of significant interactions, any influence of age, years post-disease onset and years of education was investigated by including these data as covariates into analyses of covariance (ANCOVAs). For significant differences of interest, we report means and confidence intervals (CIs) along with partial eta squared (*ηp*^2^) values as a measure of effect size (*ηp*^2^ = 0.01, 0.06 and 0.14, indicating small, medium and large effects, respectively).

### Lesion overlay maps

**Figure 2:**
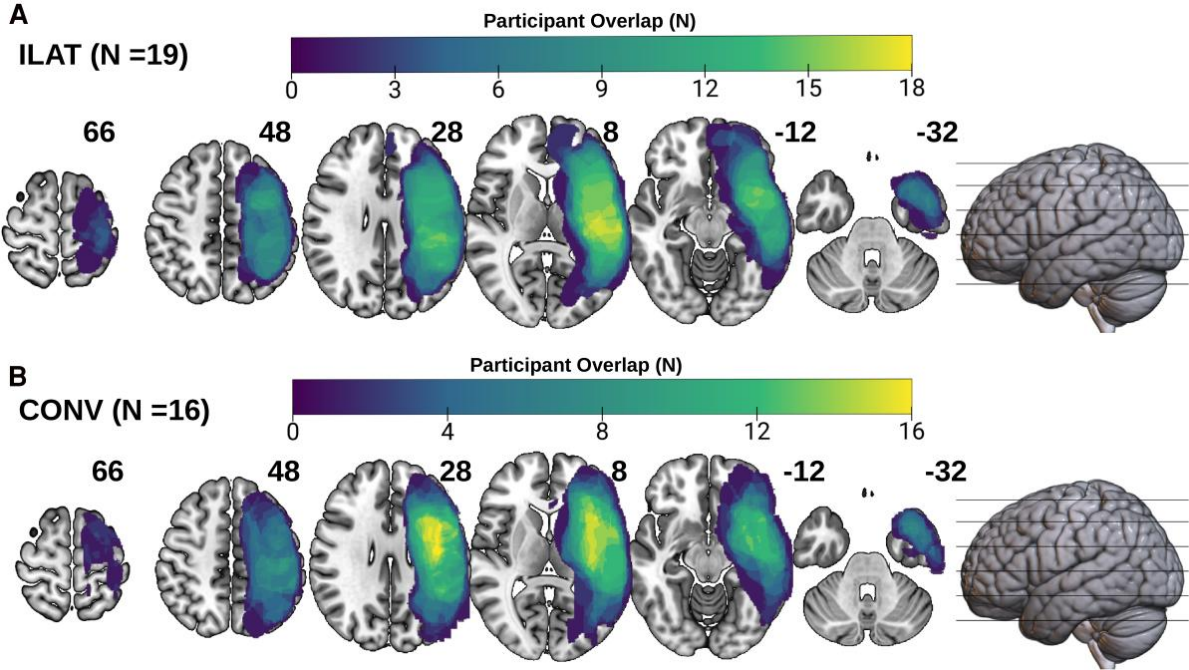
**Lesion overlap maps.** Maps for patient groups receiving ILAT (top panel) (**A**) and intensive conventional therapy (CONV; bottom panel) (**B**). Structural MRI scans were available for 19 (ILAT) and 16 (CONV) patients. The heatmap indicates the number of participants with overlapping lesions at each voxel (0–16), as shown in the colour bar labelled *Participant Overlap* (*N*; *N* = sample size). Numbers above each slice denote MNI *z*-coordinates. This visualization is descriptive and does not involve statistical testing.

Individual lesion templates were generated for each patient based on either T1 structural MRI images or CT images using MRIcroGL (https://www.nitrc.org/projects/mricrogl). These lesion maps were then normalized^63^ to MNI standard space using the clinical toolbox from SPM12.^64^ Due to missing data or image artefacts, images of nine patients (ILAT, *n* = 4; CONV, *n* = 5) could not be included in the lesion overlay maps (Fig. 2).

## Results

### Baseline difference tests

Before intervention, at time point T1, independent samples *t*-tests between therapy types were insignificant for mAAT (*P* > 0.14), BDI (*P* > 0.90), ACT (0.24), ANELT (0.89), CAL (0.13) and MADRS (*P* > 0.36) scores. Furthermore, the groups were balanced and did not significantly differ on clinical and sociodemographic variables (Table 1), including age (*P* > 0.41), sex (*P* > 0.40), time since disease onset (*P* > 0.35), years of education (*P* > 0.13) and aphasia severity (*P* = 0.27). Finally, as can be seen from Fig. 2, lesion extent and topography were comparable across groups.

### Language and communication tests

#### AAT

**Figure 3:**
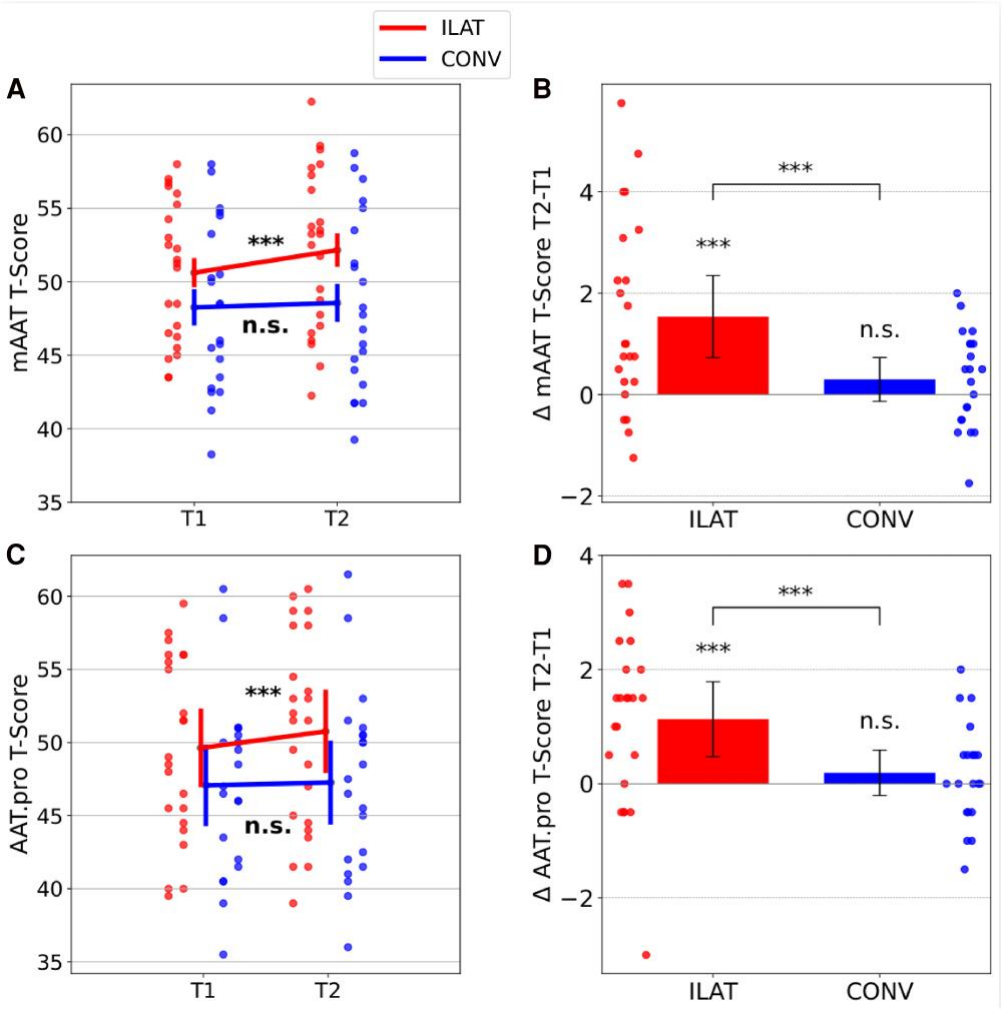
**Language outcome measures across therapy types.** Clinical language test results before (T1) and after (T2) therapy [panels on the left; (**A**) and (**C**) and their differences (Δ, right; (**B**) and (**D**)]. The panels at the top (**A** and **B**) show ANOVA results of the primary outcome language measure, i.e. AAT mean scores (mAAT) [main effect of Time [*F*(1,42) = 16.26; *P* < 0.001; *ηp*^2^ = 0.28]; Time and Therapy Type interaction [*F*(1,42) = 7.42; *P* = 0.009; *ηp*^2^ = 0.15], those at the bottom (**C** and **D**) show ANOVA results from averages of AAT subtests involving language production (subtests *Naming* and *Repetition*) {effect of Time [*F*(1,42) = 12.22; *P* = 0.001; *ηp*^2^ = 0.23]; Time × Therapy Type interaction [*F*(1,42) = 6.19; *P* = 0.02; *ηp*^2^ = 0.13]}. Results of the ILAT group (*N* = 23) are shown in red, those of the Conventional Naming Therapy (CONV) group (*N* = 21) in blue. Scatterplots show individual data points. Group means and individual data points are displayed for each time point (T1 and T2) and for pre–post differences (T2 − T1); in the line plots on the left (**A** and **C**), whiskers indicate CIs of each plotted cell, whereas in the bar plots on the right (**B** and **D**), CIs of T2 − T1 differences are shown. Asterisks mark significant changes revealed by planned-comparison paired *t*-tests (*P ≤ 0.05; **P ≤ 0.01; ***P ≤ 0.001). Positive changes indicate performance improvement. Note the significantly more substantial language improvement across ILAT as compared to CONV.

The primary outcome measure for language assessment, mAAT, revealed a significant main effect of *Time* [*F*(1,42) = 16.26; *P* < 0.001; *ηp*^2^ = 0.28] and a significant interaction between *Time* and *Therapy Type* [*F*(1,42) = 7.42; *P* = 0.009; *ηp*^2^ = 0.15], which is illustrated in Fig. 3. Planned comparison *t*-tests showed a significant decrease of mAAT scores between T1 and T2 for the ILAT group [*t*(22) = 3.93; *P* < 0.001; mean difference (*M*) = 1.54; CI = 0.73–2.35], but not for CONV [*t*(20) = 1.44; *P* > 0.17]. A follow-up ANCOVA, including age, time post-aphasia onset and years of education, failed to reveal any significant effect of these covariates on the main result (all *P*-values > 0.11).

To gain a better understanding of the factors driving the mAAT results, we conducted separate 2 × 2 ANOVA analyses on AAT production (AATpro) and comprehension (AATcom) scores. Similar to the mAAT results, AATpro scores showed a significant main effect of *Time* [*F*(1,42) = 12.22; *P* = 0.001; *ηp*^2^ = 0.23] and a significant *Time × Therapy Type* interaction [*F*(1,42) = 6.19; *P* = 0.02; *ηp*^2^ = 0.13] (Fig. 3). *Post hoc* planned comparison *t*-tests revealed that there was a significant increase of AATpro scores between T1 and T2 for ILAT [*t*(22) = −3.57; *P* = 0.002; *M* = 1.13; CI = 0.47–1.79] but not for CONV (*P* > 0.33). AATcom scores just revealed a significant main effect of time [*F*(1,42) = 6.88; *P* = 0.01; *ηp*^2^ = 0.14]. Further ANOVAs performed on the results of AAT subtests confirmed significantly stronger improvements for ILAT than conventional therapy in Naming [*F*(1,42) = 4.67; *P* = 0.04; *ηp*^2^ = 0.10], with significant difference between T1 and T2 for ILAT only [*t*(22) = −2.43; *P* = 0.03; *M* = 1.35; CI = 0.20–2.50], and a tendency towards an interaction effect on the Token Test [*F*(1,42) = 3.52; *P* = 0.07; *ηp*^2^ = 0.08], but not for Repetition (*P* > 0.11) or Comprehension (*P* > 0.32). Across therapy, planned comparison *t*-tests showed ILAT-related improvements on naming and the other three subtests, i.e. for Repetition [*t*(22)=−3.53; *P* = 0.002; *M* = 0.91; CI = 0.38–1.45], Comprehension [*t*(22) = −2.26; *P* = 0.03; *M* = 2; CI = 0.17–3.38] and Token Test [*t*(22) = −2.2; *P* = 0.04; *M* = 1.88; CI = 0.11–3.66]. For conventional utterance-centred therapy, AAT subtests failed to support significant improvements, except for a tendency (*P* = 0.07, two-tailed) on the Repetition subtest, which survived one-tailed testing.

#### ANELT

**Figure 4:**
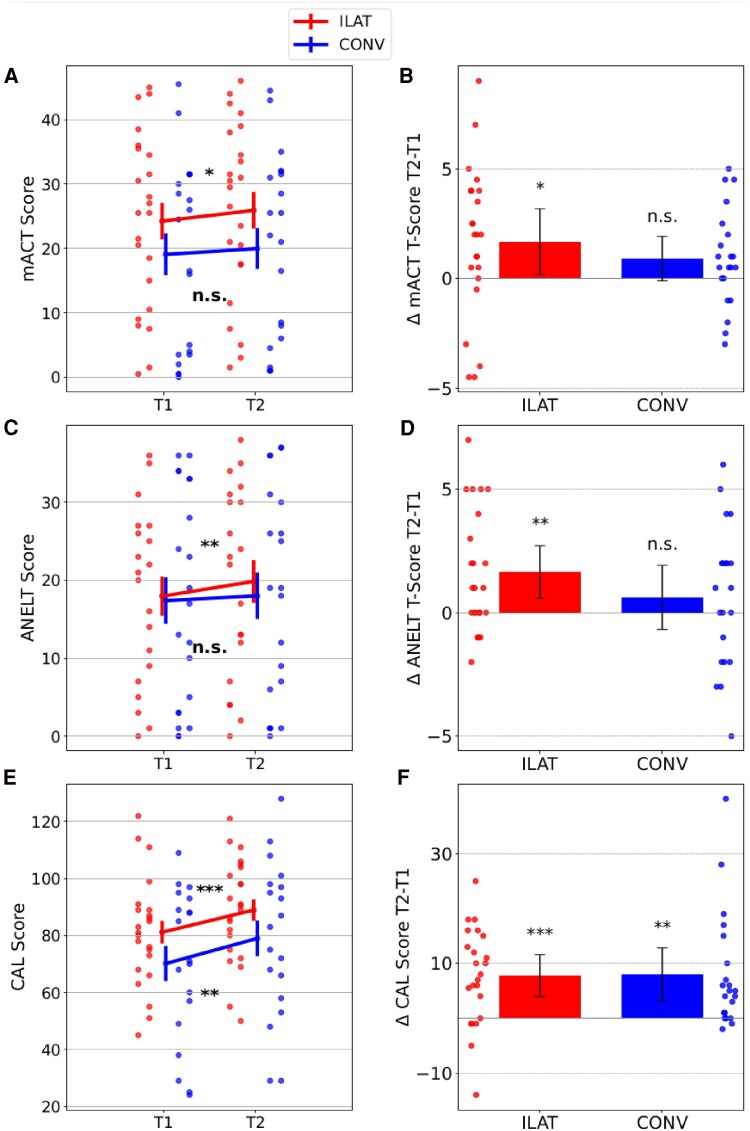
**Communication outcome measure across therapy types.** ANOVA and planned comparison results on communication and effective language use before (T1), after (T2) and across (Δ) therapy [ILAT and Conventional Naming Therapy (CONV)] according to ANELT {top, **A** and **B**; ILAT: [*t*(19) = −3.33; *P* = 0.004; *M* = 1.9; CI = 0.71–3.09]; CONV (*P* > 0.33)}, ACT {middle, **C** and **D**; ILAT [*t*(22) = −2.32; *P* = 0.03; *M* = 1.67; CI = 0.18–3.17]; CONV (*P* > 0.08)} and CAL {bottom panels, **E** and **F**; ILAT: [*t* (22) = −4.22; *P* < 0.001; *M* = 7.76; CI = 3.94–11.58]; CONV [*t*(18) = −3.58; *P* = 0.002; *M* = 8.84 CI = 3.65–14.04]}. Results of the ILAT group (*N* = 23) are shown in red and those of the CONV group (*N* = 21) in blue. Group means, individual data points and CIs are displayed as in Fig. 3. Positive changes indicate performance improvement.

Data from three participants were not obtained due to scheduling errors. The effect of *Time* [*F*(1,39) = 8.84; *P* = 0.005; *ηp*^2^ = 0.19] was significant, but the interaction did not reach significance (*P* > 0.14). Within group planned comparison *t*-tests revealed a significant improvement of ANELT scores for ILAT [*t*(19) = −3.33; *P* = 0.004; *M* = 1.9; CI = 0.71–3.09] but not for CONV (*P* > 0.33; Fig. 4).

#### ACT

The 2 × 2 ANOVA revealed a significant main effect of *Time* [*F*(1,42) = 8.47; *P* = 0.006; *ηp*^2^ = 0.17], but the interaction failed to reach significance (*P* > 0.39). Subsequent planned comparison *t*-tests showed a significant difference between T1 and T2 for ILAT [*t*(22) = −2.32; *P* = 0.03; *M* = 1.67; CI = 0.18–3.17], but not for CONV (*P* > 0.08; Fig. 4).

#### CAL

**Figure 5:**
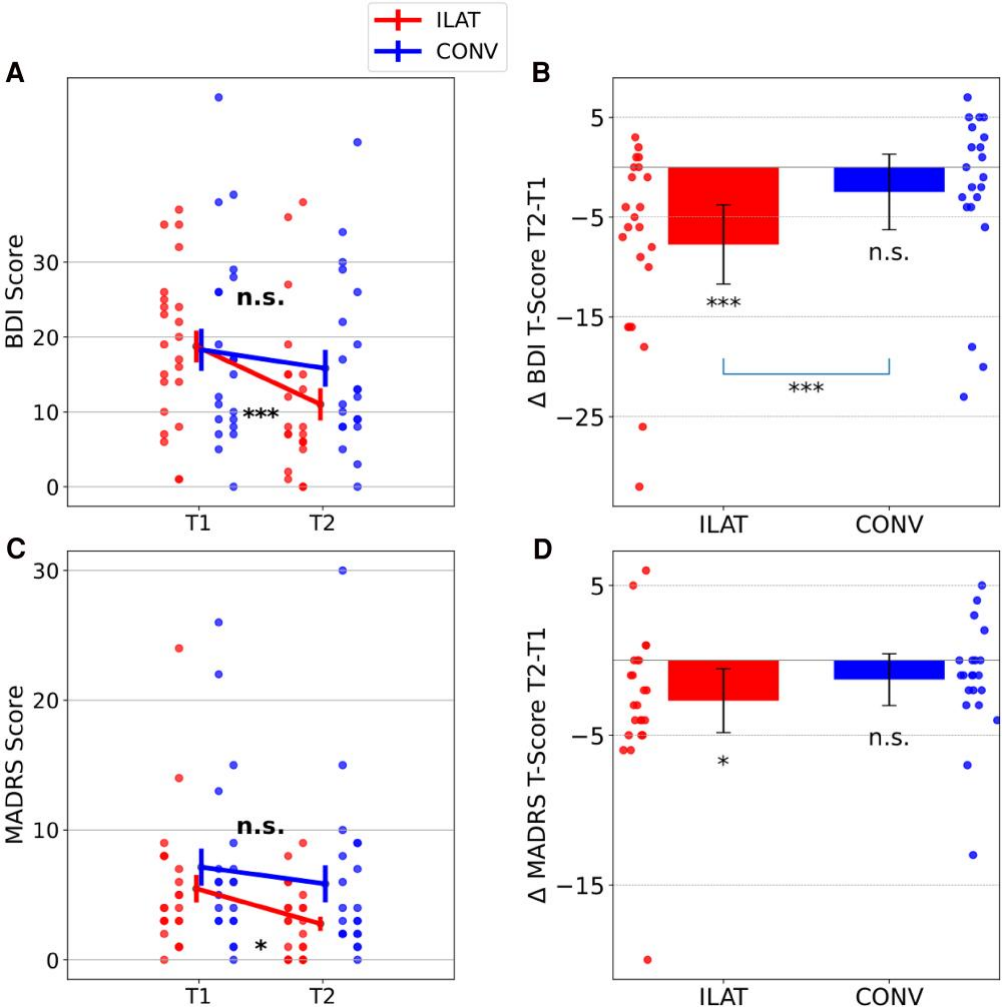
**Depression outcome measures across therapy types.** ANOVA results on the BDI [top; **A** and **B**; Time × Therapy Type interaction: *F*(1,38) = 6.94; *P* = 0.01; *ηp*^2^ = 0.15], the primary outcome measure on depressive symptoms and the MADRS [bottom panels; **C** and **D**; effect of Time: *F*(1,42) = 8.88; *P* = 0.005; CI = −12.79 to −4.54], the secondary depressiveness measure, applied before (T1), after (T2) and across (Δ) therapy [ILAT and Conventional Naming Therapy (CONV)]. Results of the ILAT group (*N* = 21) are shown in red and those of the CONV group (*N* = 19) in blue. Group means, individual data points and CIs are shown as in Figs 3 and 4. In this figure, negative changes indicate reduction of depressiveness, i.e. clinical improvement. Note the significant ILAT-specific reduction of symptoms of depression revealed by the primary outcome measure BDI.

The quantity of communication questionnaire of the communicative activity log (CAL) revealed a significant main effect of *Time* [*F*(1,40) = 30.14; *P* < 0.001; *ηp*^2^ = 0.43]. Planned comparison paired *t*-tests demonstrated a significant pre–post difference for both ILAT [*t*(22) = −4.22; *P* < 0.001; *M* = 7.76; CI = 3.94–11.58] and CONV [*t*(18) = −3.58; *P* = 0.002; *M* = 8.84; CI = 3.65–14.04] (Fig. 5).

### Depression tests

#### BDI

The primary outcome addressing hypothesis (iii) showed a significant main effect of *Time* [*F*(1,42) = 14.89; *P* < 0.001; *ηp*^2^ = 0.26] and a near-significant *Time × Therapy Type* interaction [*F*(1,42) = 3.95; *P* = 0.05; *ηp*^2^ = 0.17], which, after removal of four outliers, two from each group, using the standard criterion of lying >2 SDs from the mean (of T1/T2), yielded significance [*F*(1,38) = 6.94; *P* = 0.01; *ηp*^2^ = 0.15; Fig. 5]. Further examination showed a significant improvement for ILAT [*t*(20) = 4.38; *P* < 0.001; *M* = −8.67; CI = −12.79 to −4.54] but not for CONV (*P* > 0.25).

#### MADRS

In the analysis of MADRS scores, the 2 × 2 repeated-measures ANOVA revealed a significant effect of *Time* [*F*(1,42) = 8.88; *P* = 0.005; CI = −12.79 to −4.54]. Subsequent planned comparison paired samples *t*-tests once again indicated a significant difference between T1 and T2 for ILAT [*t*(22) = −2.62; *P* = 0.016; *M* = −2.7; CI = −4.83 to −0.56], but not for CONV (*P* > 0.13; Fig. 5).

## Discussion

This RCT of intensive SLT provided 12.5 h of treatment per week for 2 weeks to 44 chronic PSA patients. As the main result, this study revealed different clinical outcomes for the two therapy types applied, social-communicative and utterance-centred conventional SLT. The primary outcome of language performance, mAAT, showed significantly stronger improvements for the social-communicative method, ILAT, than for the utterance-centred conventional treatment, CONV, focusing on naming, picture description and related comprehension. ILAT-related improvement was manifest on all four AAT subtests applied—naming, comprehension, repetition and Token Test—whereas CONV results suggested repetition gains specifically, thus yielding some benefit too.

These results confirm that intensive SLT is effective in chronic PSA [hypothesis (i)]. However, most importantly, they also show that the amount and intensity of SLT are not the feature of SLT determining its effectiveness. Amongst intensive SLT methods, the communicative function with which language is used during therapy may substantially modulate therapy success. Involving patients in game-like requests and planning conversations during SLT using the ILAT method yielded significantly better language outcomes than delivering the same amount and intensity of naming, picture description and related comprehension training. A reason for this difference in effectiveness may lie in the motivation and reinforcing role of communication and of the experience of realizing one’s own communicative goals within the therapy context. We will discuss this point in more detail below.

Interestingly, the primary measure of symptoms of depression, the BDI, mirrored the language-related effects by revealing—after removal of outliers—a significant interaction due to improvements of mood, which may result in enhanced quality of life. This effect was seen specifically in PSA patients undergoing social-communicative ILAT, but not in those receiving conventional treatment. This result may indicate that either the language improvement itself or features of the SLT method applied had a positive effect on patients’ mood.^41^

### Different benefits of high-intensity SLT methods with matched materials and linguistic forms

The primary outcome measure, a language performance score calculated from four subtests of the Aachen Aphasia Test (mAAT), demonstrated stronger therapy-induced improvements for the social-communicative method, ILAT, than for the intensive conventional treatment focusing on naming, picture description and related comprehension. This difference between communicative and conventional-structural therapy was shown by a significant interaction of the factors Time and Therapy Type, with across-treatment improvements for ILAT, but only marginal change for the conventional SLT method. The speech production component of the AAT calculated from naming and repetition subtests fully confirmed this pattern of results, suggesting that any differences in therapy benefit are primarily manifest in speaking. Furthermore, the pattern was partially explained by performance changes on the Naming and, possibly, Token Test subtests.

The main result of this study is that the same amount and intensity of two different behavioural SLT methods, performed as group treatments, can lead to significantly different outcomes. However, this finding contrasts with statements in current reviews,^1,65^ emphasizing the absence of evidence for one type of intensive behavioural SLT being superior to other such treatments. This should particularly be true if, as in the current study, not only the amount and frequency of the compared treatments were identical across therapy types, but also the same materials and picture cards were applied and the same target utterances were practised. Note again that, in the present work, much care was spent to apply the same sets of picture cards across both types of therapies and that these picture cards similarly constrained the target utterances to be produced and understood by the participants. Furthermore, these picture cards did not contain items used for testing, so that trivial therapy effects due to training of test utterances are excluded. In addition, the patient groups did not differ significantly with respect to age, time since disease onset, level of education or aphasia severity, and their lesion profiles were comparable (Fig. 2). Moreover, both methods were applied in groups of three patients and one therapist.

We present evidence that two different but well-matched aphasia therapy methods—one conventional and the other social-communicative—yield different non-trivial positive effects on language and mood in chronic PSA, in spite of being applied with the same intensity, using the same materials in patients with similar aetiology and aphasia severity who worked together in groups of the same size. This finding demonstrates that a feature unrelated to the amount and frequency of therapy was crucial for therapy efficacy.

### Communicative language use as a critical factor

That social-communicative treatment of aphasia may be advantageous over utterance-centred, structural conventional language training has frequently been suggested,^13-16^ but insufficient evidence from RCTs had been available in support of this proposal.^12,25,27,28,29,66^ Some support was provided by our 2001 RCT comparing equal amounts of conventional therapy to CIAT,^1^ the ILAT pre-curser, suggesting better outcomes for the latter. However, this result was subject to confounding by therapy frequency, as the overall 30 h of therapy had been offered in time frames of 15 versus 7–8 h per week. More recent evidence shows that, although both therapy frequency and amount are important factors,^5,6^ increasing intensity substantially above a level of 5 or 6 h per week remains without additional benefit,^9^ thus making it unlikely that the intensity difference of 15 versus 7–8 h per week explains the better outcome of the communicative method in the aforementioned study. In fact, a further pilot study found different outcomes of intensity-matched conventional and communicative therapies in a small number of patients,^32^ which our present results now extend and solidify. These earlier studies already suggested what this work now shows, that using language in social-communicative context of language games leads to an efficacy advantage over conventional utterance-centred training of linguistic structures *per se*.

The embedding of language in communicative social contexts as it is realized in CIAT and ILAT has different features, each of which could be relevant for the observed therapeutic benefit. In contrast to naming, which is typically followed by therapeutic judgement and reinforcement, a range of different responses regularly follow the requests or proposals immanent to communicative language games. For example, requests invite (i) the requested non-linguistic action; (ii) request rejection if the matching card is unavailable; and (iii) repair sequences in case of difficulty in understanding. Therefore, conventional and communicative therapy differ (i) in their richness of language use. As an additional difference, interactions in utterance-centred training are primarily between patient and therapist, whereas the communicative method is characterized by continuous alteration of interacting partners. These (ii) changes of dialogic dyads are more attention and memory demanding than the constant pairing of conventional therapy, because more potential partners and their background knowledge need to be taken into account. Furthermore, production and understanding of a target word or utterance are the sole aim of conventional therapy, whereas language games allow for realizing one’s own communicative goals by way of utterance usage. Therefore, a further difference addresses (iii) the positive reinforcement resulting from successful social-communicative interaction, which, in the case of ILAT, adds to reinforcement given by the therapist (a feature shared by both methods). All these distinguishing features—richness of language use, alteration of dyads and social reinforcement—may specifically or jointly contribute to the observed efficiency advantage of ILAT over conventional therapy. Since many chronic aphasia patients severely suffer from the loss of social interaction and contact brought about by their language deficit, we hypothesize that the experience of social-communicative success in therapy might be the most important factor driving therapeutic progress.

The present results were obtained with specific conventional and communicative SLT methods but may generalize to closely related treatments. In particular, the effects of intensive conventional therapy here applied to groups of three patients may be similarly present in intensive 1:1 therapy in which picture naming and description and their understanding represent a main component. Similarly, communicative group treatment results may generalize to communicative 1:1 treatment. As the ILAT method uses language games already established in CIAT, these methods may yield similar outcomes. CIAT constrains communication to verbal language, whereas the ILAT method applied here did not, because earlier studies showed similar outcomes with and without the ‘verbal-only’ constraint.^33,59^ Similarly, the communicative method known as Promoting Aphasics’ Communicative Effectiveness (PACE^14,28^), which focuses on informing as the main speech act trained in a game-like context, may likely yield comparable results (see, e.g.^33,59,67^), similar to other intensive and communicative therapeutic perspectives developed in the context of CIAT or ILAT, as, for example the Intensive Comprehensive Aphasia Program.^68^ However, these hypotheses need further testing in RCTs.

### Results of communication tests

The communication tests ANELT and ACT indicated that the significant language performance improvements in ILAT participants were also manifest in day-to-day social interaction (ANELT, *P* = 0.002; ACT, *P* = 0.026), whereas comparable effects were absent or just approached significance in the CONV group (ANELT, *P* > 0.3; ACT, *P* = 0.06). These results may suggest that ILAT-based communicative SLT has benefits in day-to-day social interaction of patients, although we hesitate to add any strong interpretation of these results as the relevant interactions were statistically non-significant. The Communicative Activity Log suggested improvements for both treatment types. However, we note that the quantity-of-communication part of the CAL was evaluated by patients’ relatives most of whom also were heavily engaged in daily transport of patients to the therapy facility and spent substantial time travelling with the patient and waiting during therapy each day. This may have created an expectation bias which might have affected their ratings on the CAL.

### Effects on symptoms of depression

The primary outcome measure for assessing symptoms of depression, BDI, revealed a borderline interaction effect of Time and Therapy Type, which, after removal of outliers, yielded significance. Planned comparison tests showed an improvement across ILAT but not for conventional therapy. Likewise, the MADRS, revealed significant improvements of depression scores for the ILAT but not the conventional therapy cohort.

These results are in line with earlier findings of reduced symptoms of depression brought about by the ILAT method.^41-43^ Successful participation in behaviourally relevant social-communicative interaction therapy appears to benefit patients’ mood, quality of life and possibly functional recovery more generally, an effect not seen for conventional language practice. This observation provides one more argument for the relevance of intensive communication therapy in chronic PSA. It also raises the question whether any changes in mood and depressive symptoms are a consequence of the improved communicative effectiveness the patients experience across therapy. However, further analyses failed to document a statistically significant correlation between patients’ language improvements and reduction of depressive symptoms as revealed by this trial’s respective primary outcomes. This observation discourages the hypothesis of an origin of mood changes solely in linguistic or communicative improvements. Nevertheless, it is consistent with the result of two out of three communication measures (CAL, ACT) that ILAT, but not conventional therapy, may improve communicative-pragmatic skills. This communicative benefit could have positively influenced patients’ symptoms of depression by increasing their self-efficacy via their enhanced communicative-pragmatic skills. Alternatively, aspects of intensive communicative interaction implemented in ILAT may have impacted on symptoms of depression directly, without the mediation of language. The causal chain leading from the applied social-communicative intervention to the improvement of the patients’ depressive symptoms thus awaits clarification in future studies.

## Data Availability

Anonymized data are available from the authors upon reasonable request.
